# Compound cancer with small cell carcinoma and mucinous adenocarcinoma of the ovary: a case report and literature review

**DOI:** 10.3389/fonc.2025.1500088

**Published:** 2025-02-25

**Authors:** Wan Peng, Hui Hu, Genhua Huang, Xiaoyun Wu, Yichen Zhu, Tian Yang, Xin Tao, Buzhen Tan

**Affiliations:** ^1^ Department of Obstetrics and Gynecology, The Second Affiliated Hospital, Jiangxi Medical College, Nanchang University, Nanchang, China; ^2^ Department of Pathology, The Second Affiliated Hospital, Jiangxi Medical College, Nanchang University, Nanchang, China

**Keywords:** ovarian cancer, small cell carcinoma, mucinous adenocarcinoma, gynecologic tumor, case report

## Abstract

Primary small cell carcinoma of the ovary is an extremely rare and highly malignant ovarian malignancy. The tissue origin of this type of cancer is still unclear, and it is characterized by rapid progression and a discouraging prognosis. The clinical presentation of small cell carcinoma of the ovary lacks specificity, and there are currently no effective treatment options available. Therefore, it is crucial to improve the understanding and identification of this disease. In this paper, we present a case of composite small cell carcinoma of the ovary with mucinous adenocarcinoma and provide a comprehensive review of the relevant literature.

## 1 Introduction

Ovarian cancer is one of the three primary malignant neoplasms that affect the female reproductive system, with compound ovarian cancer constituting a rare presentation that accounts for fewer than 1% of all ovarian tumors (1). A mixture of small cell carcinoma and mucinous carcinoma of the ovary is even more rarely observed, and only seven cases of this unique type of compound carcinoma have been reported in the literature in both China and internationally (2–8). In the 2014 edition of the World Health Organization (WHO) Classification of Tumors of the Female Genital Organs, small cell carcinoma of the ovary (SCCO) is categorized as a miscellaneous ovarian tumor due to its unclear histologic origin. Based on clinical and pathologic characteristics, SCCO can be further divided into two subtypes: ovarian small cell carcinoma of the hypercalcemic type (SCCOHT) and ovarian small cell carcinoma of the pulmonary type (SCCOPT) (9). The 2020 edition of the WHO Classification of Tumors of the Female Genital Organs then reclassified small cell carcinoma of miscellaneous ovarian tumors into two categories: the hypercalcemic type and the large-cell variant. Furthermore, in this updated classification, SCCOPT was incorporated into the discussion of neuroendocrine tumors for the first time (10). We herein describe a rare case of compound ovarian cancer, i.e., small cell carcinoma with mucinous adenocarcinoma of the ovary. Considering its clinical significance, we believe that it merits further attention.

## 2 Case description

Our case involved a 24-year-old young woman with no previous history of pregnancy or marriage. Her menarche was at 12 years of age, and her menstrual cycle was regular, with no evidence of menorrhagia or dysmenorrhea. Her medical history included anxious depression for 3 years, and she was under treatment with oral trazodone hydrochloride. Other than this, she had no remarkable past medical history and did not reflect a family history of cancer. She was referred to our hospital on 20 July 2023 due to abdominal pain and distension for 1 week and the discovery of a pelvic mass. The abdomen was distended under full abdominal pressure with no rebound pain. On internal examination, a full-term pregnant uterine mass could be palpated in the pelvis, and there was tension, a clear demarcation, and tenderness.

The results of the tests conducted in the laboratory were as follows: a blood calcium of 2.22 mmol/L; serum cancer antigen 125 (CA125) of 334.10 U/ml; serum alpha-fetoprotein, 35.1 ng/ml; and human epididymal protein 4 (HE4), 84 pmol/L (normal ranges, 2.11–2.52 mmol/L, 0–35 U/ml, 0–8.1 ng/ml, and 29.25–68.50 pmol/L, respectively). Routine blood tests showed the following: white blood cell count, 11.03×10^9^/L; red blood cell count, 3.67×10^12^/L; hemoglobin, 105 g/L; platelet count, 346×10^9^/L; neutrophilic granulocyte percentage, 78.7%; lymphocyte percentage, 13.5% (normal ranges, 3.5–9.5×10^9^/L, 3.8–5.1×10^12^/L, 110–150 g/L, 125–350×10^9^/L, 40%–75%, and 20%–50%, respectively).

**Figure 1:**
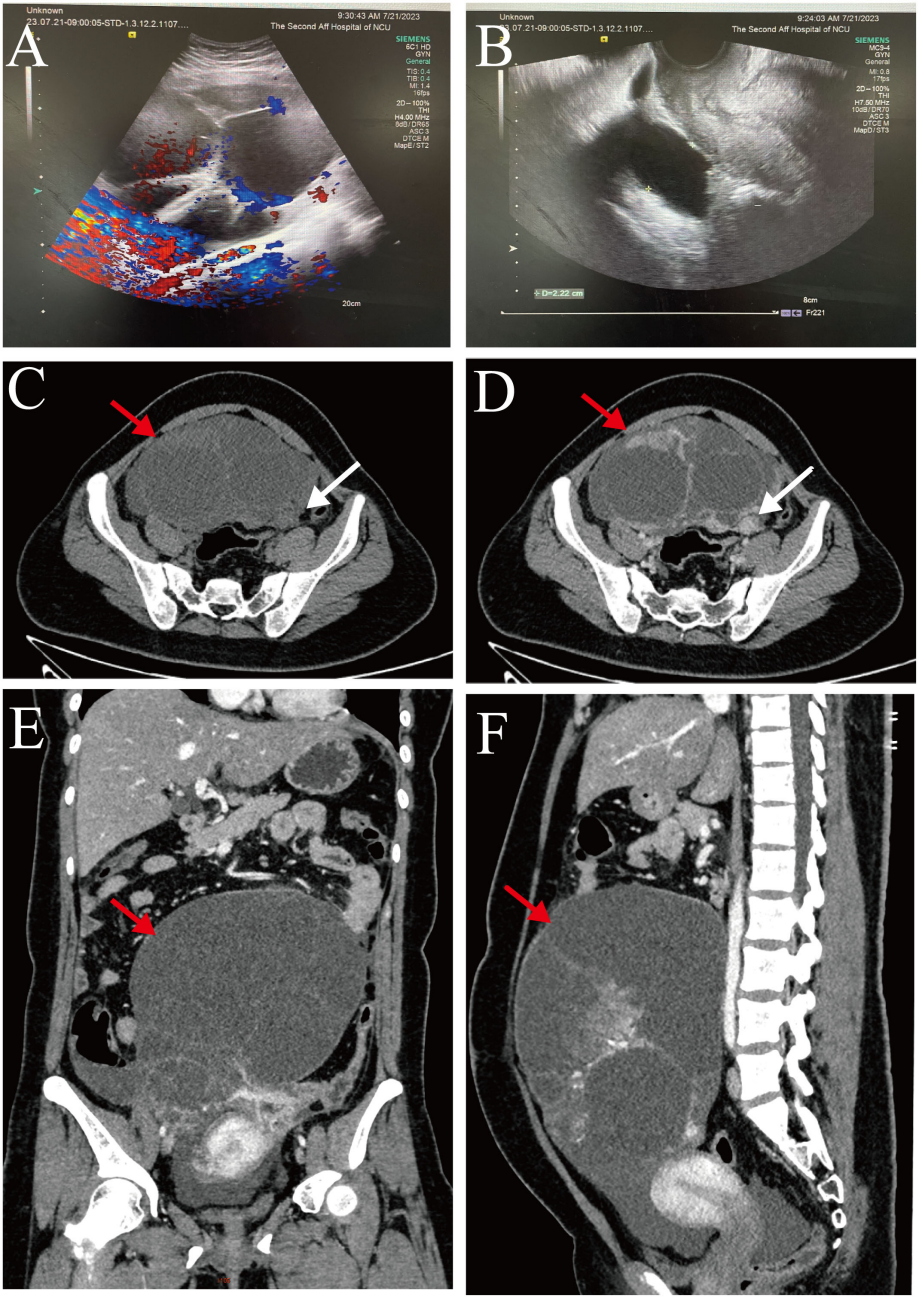
**(A, B)** Transvaginal color ultrasound scan: The left ovary showed unclear and there was a predominantly cystic mixed echogenic mass in the lower abdomen (size about 40 cm × 45 cm × 15 cm). **(C-F)** Whole-abdomen enhanced computed tomography images: There was a large cystic solid mass (red arrow) in the abdominopelvic cavity that was in close proximity to the left adnexa (white arrow).

Transvaginal color ultrasound scan showed a cystic, predominantly mixed echogenic mass with multiple septations, and a small blood flow signal was detected on the septations in the lower abdomen that measured approximately 40 cm × 45 cm × 15 cm (**Figures 1A, B**).

Whole-abdomen enhanced computed tomography showed large cystic solid occupations in the abdominopelvic cavity (size, approximately 135 mm × 189 mm), and cystic solid tumor of left adnexal origin was considered. We also noted thickening of the omentum and the peritoneum and accumulation of fluid in the abdominopelvic cavity. In addition, we observed bilateral pleural effusion, a small amount of pericardial effusion, and several lymph nodes in the right cardio phrenic angle (**Figures 1C–F**).

**Figure 2:**
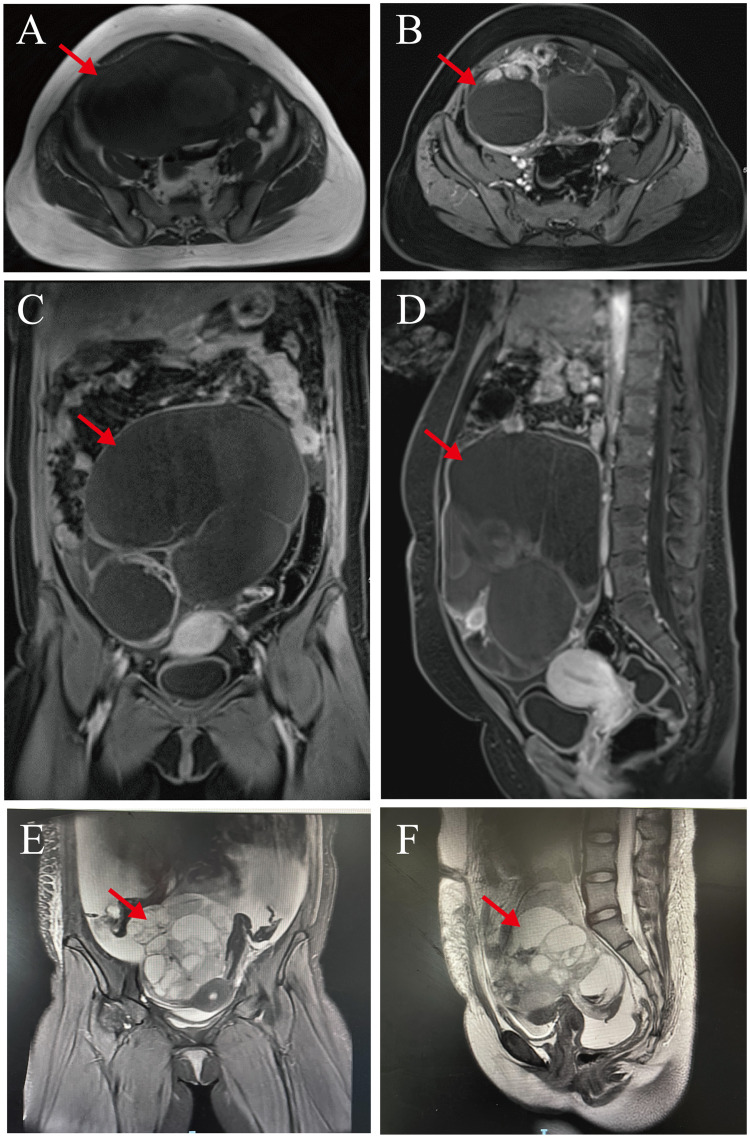
Pelvic magnetic resonance images. **(A–D)** Preoperative pelvic magnetic resonance images: Abdominopelvic cavity shows a large, cystic solid mass. Solid and septal portions of the lesion show marked inhomogeneity that was in close proximity to the left adnexa (red arrows). **(E, F)** Three month postoperative pelvic magnetic resonance images: There was a large cystic-solid mass (cross-sectional size of about 122 × 95 × 116 mm) in the pelvis(red arrows), and the adjacent structural tissues were displaced by its compression.

Pelvic magnetic resonance (MR) imaging revealed a large, cystic solid mass in the abdominopelvic cavity with long T1 and T2 signals and multiple linear segregations, with the solid portion of the lesion manifesting slightly shorter T1 signals and slightly longer T2 signals. The solid portion of the lesion and the segregation showed obviously uneven enhancement that was most closely juxtaposed to the left adnexa. Our impression was of a large, cystic-solid occupancy in the abdominopelvic cavity, and we considered it to be a cystic-solid tumor of left adnexal origin that suggested malignancy when assessed using tumor indicators. We also noted thickening of the omentum and peritoneum and abdominopelvic effusion (**Figures 2A–D**).

## 3 Intraoperative exploration

**Figure 3:**
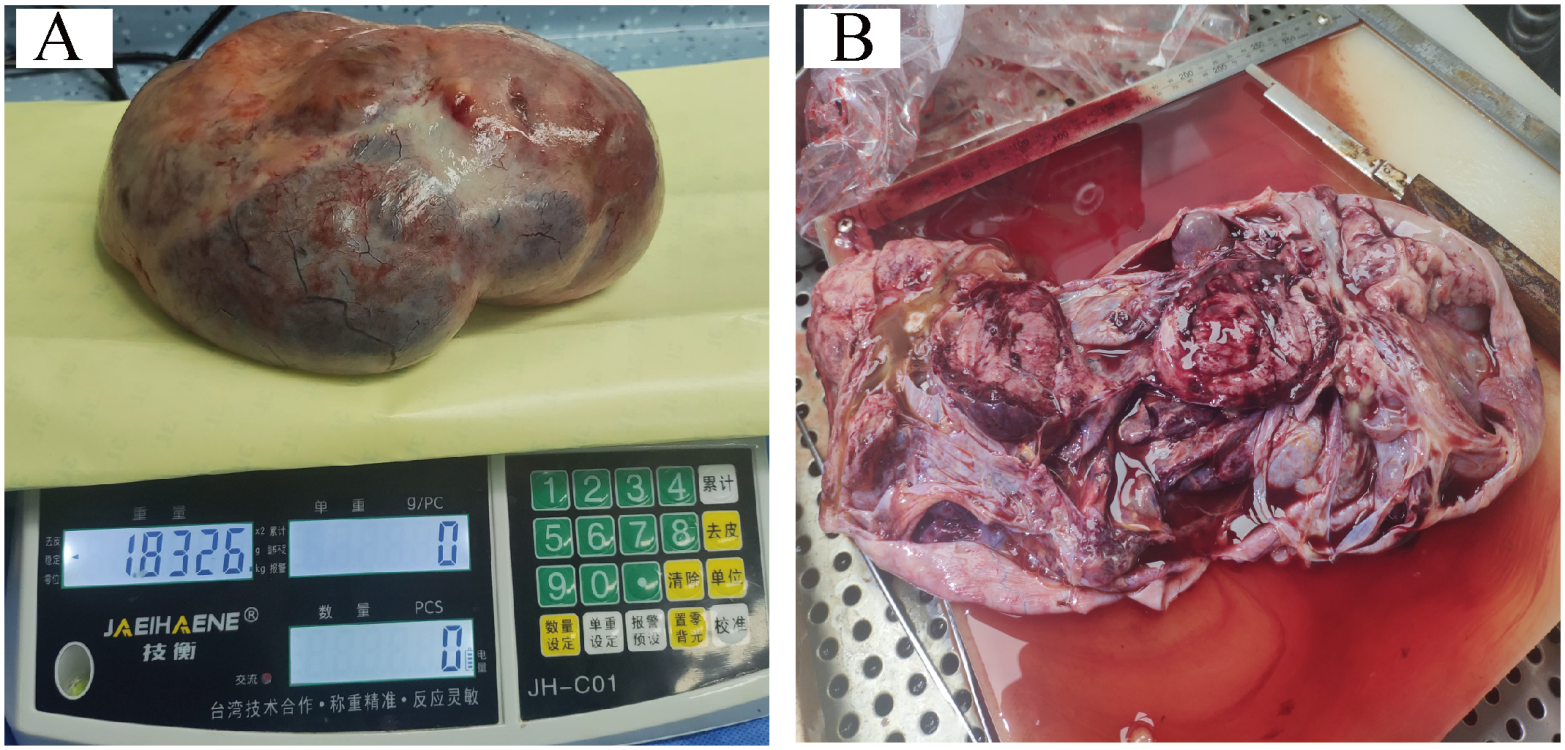
**(A, B)** Post-tumor resection specimen.

The patient was suspected of having an ovarian malignancy, so she underwent an exploratory laparotomy on July 24, 2023 under general anesthesia. A large, cystic solid mass was observed in the pelvis, measuring approximately 40 cm × 45 cm × 30 cm, which was partially cystic and partially solid, with dense adhesions to the mesentery and greater omentum. After the separation of the adhesions, the mass appeared to have originated from the left ovary. The right ovary was inflammatory and congested, and there was no obvious abnormality in either fallopian tube. The mass and the left adnexa were completely resected and the pelvic and abdominal cavities were explored. We found no lesions in the uterus, the right adnexa, the left fallopian tube, the pelvic wall, the abdominal wall, the greater omentum, the intestinal surface, the liver, the spleen, or the stomach. Finally, we measured the weight of this tumor to be about 1.8326 kilograms (after aspiration of 1300 mL of intracapsular fluid) (**Figures 3A, B**). Rapid intraoperative pathology indicated a malignancy in the left ovary. The ovarian tumor was ruptured during surgery, and we thus described it as stage IC1. The patient’s family stated that the young woman was unmarried and childless and that they wished to preserve her fertility and not to expand the scope of surgery. Therefore, only a left salpingo-oophorectomy was performed.

## 4 Postoperative pathology

**Figure 4:**
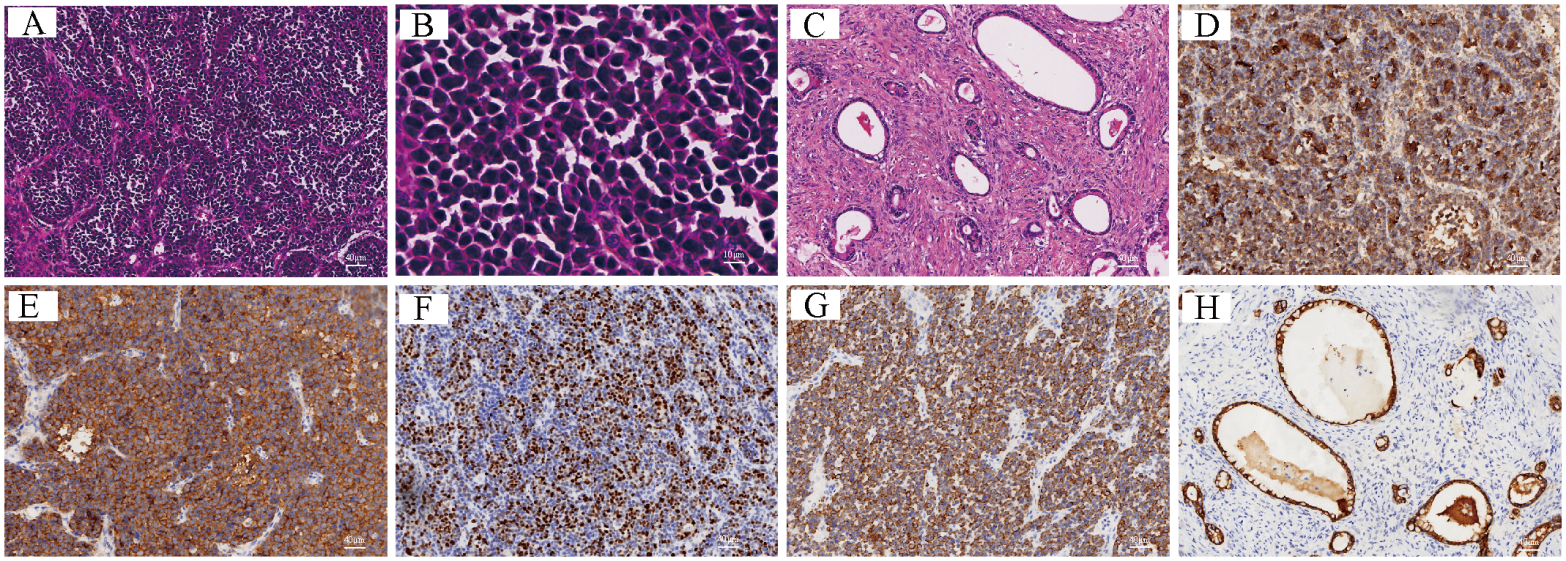
**(A–H)** Histopathological and immunohistochemical images: **(A, B)** Small cell carcinomas: tumor cells are organized in clusters or pseudopapilllae with well-stained nuclei (**A**: H&E, x100; **B**: H&E, x400); **(C)** Mucinous adenocarcinoma: mucus is seen within the cells, and the cells are arranged in an adenoidal pattern (H&E, x100); **(D–H)** immunohistochemical features of the tumor (EnVision, x100): Small-cell carcinoma was positive for CgA **(D)** and Syn **(E)**, for Ki-67 expression at approximately 40% **(F)**, and for cytokeratin **(G)**; mucinous adenocarcinoma was positive for cytokeratin **(H)**.

Macroscopic findings of the left ovary and tumor specimen showed a nodular mass that measured 23.5 cm × 17.5 cm × 8 cm, was cystic solid in section, contained bloody fluid, showed solid areas in the form of nodules, and was 1.5–5.5 cm in diameter, grayish-yellowish-grayish-red, and soft. The fibrous cystic wall was lined with monolayer high columnar epithelium microscopically, and cellular heterogeneity was observed in some areas. Two tumor cell types were noted in the solid area, with cells observed in a nested sheet or pseudopapillary arrangement in the majority of the area; some cells manifested fine nuclear staining, and some cells possessed large nuclei with eosinophilic nucleoli. The cells were arranged in an adenoidal pattern in a small portion of the area, and intracellular mucus, hemorrhage, and necrosis were seen. There were no neoplastic changes in the left fallopian tube. Immunohistochemical staining showed small cell carcinoma cells that were cytokeratin (+), chromogranin A (+), synaptophysin (+), CA199 (+), Pax8 weakly (+), P53 wild-type expression, and Ki-67 about 40% (+). CEA, CK7, Vim, SALL4, OCT4, AFP, CD117, CD30, S100, uroplakin, WT-1, P16, inhibin, calretinin, and EMA were (-). The adenocarcinoma cells were cytokeratin (+), chromogranin A partially (+), synaptophysin partially (+), CEA (+), CK7 partially (+), EMA (+), and Pax8 (+). The P53 showed wild-type expression, with CD30 scattered weakly (+), and the Ki-67 was at about 40% (+). CA199, Vim, SALL4, OCT4, AFP, CD117, S100, uroplakin, WT-1, P16, inhibin, and calretinin were (-). Ascites cytology exhibited no malignant tumor cells, and no abnormal ploidy cells were seen. Upon histopathologic examination, we determined that the excised specimens contained both small cell carcinomas and mucinous adenocarcinoma (**Figures 4A–H**).

## 5 Follow-up after surgery

The patient refused to continue treatment such as adjuvant chemotherapy or radiotherapy after the operation and went home to receive local traditional Chinese medicine (TCM) treatment. However, only 2 months after the operation, she developed abdominal distension and pain as well as chest tightness and dyspnea. Later, she underwent a Computed tomography (CT) scan at the local hospital because of increased abdominal distension. The results showed that she had right-sided pneumonia with a large amount of fluid in the right thoracic cavity and a small amount of fluid in the left thoracic cavity. And she had multiple round-like soft tissue-like masses with peritoneal fluid and omental thickening in the right abdominal cavity. After a week, her symptoms were still not relieved, so she was transferred to our hospital on October 11, 2023 for treatment. We supplemented her with a pelvic magnetic resonance (MR) examination, which showed a large mixed cystic-solid mass in her pelvis with a cross-section of approximately 122 × 95 × 116 mm, and the structural tissues around the mass were compressed and displaced as a result. The cystic wall and solid portion showed marked heterogeneous enhancement, and the subcutaneous soft tissues of the abdominopelvic wall appeared swollen. She had fluid accumulation in the abdominal and pelvic cavities, thickened and rough peritoneum, and localized nodular changes in the peritoneum (**Figures 2E, F**). Therefore, we considered that she might have tumor recurrence as well as peritoneal metastasis. After we treated her with symptomatic supportive therapy such as oxygen, anti-infection, drainage by thoracentesis and abdominal puncture, the patient’s symptoms were slightly relieved. Our multiple disciplinary team (MDT) also discussed her condition and subsequent treatment modalities, and originally planned to perform surgery after her condition improved, but the patient and family ultimately gave up the treatment. She eventually died on June 9, 2024 due to the deterioration of her condition. From the date the disease was first detected, she survived for only about 11 months.

## 6 Discussion

It is extremely rare to have both mucinous adenocarcinoma and small cell carcinoma of the ovary. To our knowledge, including the present Case Report, this compound ovarian cancer has only been reported eight times thus far (**Supplementary Table 1**) (2–8).

Previous studies have indicated that SCCOHT exhibits a higher prevalence among young women, typically manifesting at an average age of 24 years. In contrast, SCCOPT is predominantly observed in middle-aged and older women, with an average age at onset of 54 years (11, 12). The clinical manifestations of SCCO lack specificity. SCCO is commonly characterized by symptoms such as abdominal pain and distension, pelvic mass, and pressure on adjacent organs. In addition, acute abdominal symptoms can arise due to torsion or rupture of the tumor, as well as irregular vaginal bleeding (11, 12). Approximately two-thirds of patients with SCCOHT exhibited elevated blood calcium levels, with a minority of patients experiencing symptoms related to hypercalcemia including nausea, vomiting, irritable thirst, and polyuria. These symptoms may be attributed to the paracrine action of the tumor cells that generates parathyroid hormone-related protein (PTHrp) (13). Conversely, SCCOPT does not parallel hypercalcemia, and its prognosis is inferior to that of SCCOHT. SCCOHT and SCCOPT generally exhibit a negligible variation in morphology, with unilateral ovarian involvement and a large tumor size. The tumors are principally solid but often accompanied by various levels of cystic degeneration, hemorrhage, and necrosis. Additionally, the majority of affected patients exhibit elevated serum CA125 levels (14). Our patient was a 24-year-old individual who exhibited symptoms of abdominal pain and distension. The localization of the tumor was identified within the left ovary, and the tumor measured approximately 40 cm in size. Morphologically, the tumor showed a cystic solid structure that was accompanied by evident hemorrhage and necrotic areas, and this was consistent with the findings reported in the extant literature. Although the patient’s pre-operative blood calcium level fell within the normal range, elevated serum levels of CA125, AFP, and HE4 were observed. It is important to note that while blood calcium levels reflect some diagnostic value in the identification of SCCOHT, its specificity remains limited. Therefore, definitive confirmation of the diagnosis relies upon subsequent pathologic examination and immunohistochemical analysis.

The hypercalcemic type Is one where the tumor cells are manifest as follicle-like lumens of varying sizes and irregular shapes, and the lumens contain eosinophilic gelatinous material. The tumor cells are primarily composed of rounded or ovoid clusters of small cells that can be arranged in sheets, islands, strips, or beams with little cytoplasm, and they exhibit small, dark-stained nuclei with active nuclear schizophrenic phenomena. SCCOHT and malignant rhabdomyoid tumors (MRTs) exhibit morphologic similarities (15). Both types of tumors exhibit large eosinophilic cells with ample cytoplasm, noticeable nucleoli, and vacuolated nuclei. SCCOPT resembles small cell neuroendocrine carcinoma of the lung and is characterized by small tumor cells that are arranged in sheets, tight nests, islands, or beams (12). The cells have a fine pretzel-like nuclear chromatin, sparse cytoplasm, and inconspicuous nucleoli, and they frequently exhibit nuclear schizophrenia. Follicle-like structures and large cellular components are rarely seen in the tumor tissue. Immunohistochmical staining of SCCOHT tumor tissues frequently demonstrates the expression of EMA, cytokeratin, and calretinin, while typically lacking α-inhibin, S100, desmin, and thyroid transcription factor-1. In contrast, immunohistochemical staining of SCCOPT tumor tissues generally shows positive staining for chromogranin A, CD56, synaptophysin, and neural-specific enolase. SCCOPT also shows expression of EMA and cytokeratin, but α-inhibin is rarely detectable. Recent research has shown that over 90% of SCCOHT cases are associated with mutations in the SMARCA4 geneand that these mutations can result in the loss of BRG1 protein expression. The absence of expression of both SMARCA4 and SMARCA2 has also been identified as a highly accurate and specific indicator for the diagnosis of SCCOHT (16). In our case, chromogranin A and synaptophysin were positive, and cytokeratin and P53 were expressed; while EMA, calretinin, inhibin, and S100 were not expressed, suggesting a diagnosis of SCCOPT. Because our immunohistochemical results proved that the patient had ovarian small cell carcinoma complicated with mucinous adenocarcinoma, and CD56, BRG1, INI-1, TFF-1, and SOX-2 were only suggested to be expressed in some literatures, we did not test the immunohistochemical results for CD56, BRG1, INI-1, TFF-1, and SOX-2. Due to the lack of availability of further genetic testing and an overlap in histologic presentation and immunohistochmical localization between SCCOHT and SCCOPT we were not confident in distinguishing between them. In terms of tissue origin, SCCO is similar to ovarian epithelial tumors, ovarian gonadal mesenchymal tumors, and germ cell tumors. Furthermore, it is important to differentiate SCCO from metastases of neuroendocrine carcinomas originating in the lungs or other organs (12, 13).

SCCO has an extremely poor prognosis, with most patients dying or relapsing within a year or two. The prognosis of SCCO is worse than that of ovarian mucinous adenocarcinoma, and thus, the prognosis of this composite cancer depends more on SCCO. Out of the eight reported patients, five succumbed within a period of 10 months, with the majority exhibiting signs of metastasis, particularly in the peritoneum and liver (2–4, 7, 8). Our patient also experienced tumor recurrence and metastasis within 3 months after surgery and died less than 11 months later. It is highly advantageous to have an early FIGO stage, an age of at least 30 years, a normal preoperative calcium level, a tumor diameter of over 10 cm, and few large cellular components when it comes to SCCOHT (11). There is presently no standard treatment for SCCO, with surgery and chemotherapy being the primary treatments. The surgical treatments include total hysterectomy, salpingo-oophorectomy, appendectomy, and full pelvic and para-aortic lymphadenectomy. Furthermore, tumor cytoreduction surgery is feasible for patients with intermediate-to-advanced disease (16, 17). Due to the high malignancy of the disease and poor prognosis, it is controversial as to whether young women can preserve their fertility if they contract it. While postoperative adjuvant chemotherapy is crucial to prolonging patient survival, there is currently a paucity of standardized chemotherapeutic regimens. Commonly used treatments include combinations of platinum and etoposide (e.g., BEP, VPCBAE, and PAVEP) and combinations of platinum and paclitaxel. Multimodal radiotherapy can be administered after chemotherapy, but its effectiveness is unclear. Some guidelines recommend the use of high-dose chemotherapy (HDC) and autologous stem cell transplantation (ASCT) to prolong survival in patients who achieved a complete response (CR) by cytoreduction surgery or 4–6 cycles of chemotherapy (18). In addition, epigenetic therapies, kinase inhibitors, and immunosuppressive drugs are also effective against SCCO (13). Although postoperative adjuvant therapy has a prognostic impact, our patient ultimately opted out of these treatment modalities.

## 7 Conclusions

In conclusion, SCCO combined with mucinous ovarian cancer is extremely rare, and due to the lack of specificity in its clinical and pathologic manifestations, it is easily misdiagnosed as another type of ovarian tumor. Current treatment options for this disease remain limited, as conventional treatments such as surgical resection and chemotherapy are not always effective for this rare tumor type. Further research is therefore needed to uncover more precise and effective treatments. It is crucial for clinicians and pathologists to improve our understanding and identification of this disease. We posit that early diagnosis and accurate pathologic analysis will facilitate the development of individualized treatment plans and improve patient survival and the quality of life of patients.

## Data Availability

The original contributions presented in the study are included in the article/**Supplementary Material**. Further inquiries can be directed to the corresponding author.
