# Supplementary material for: Compound cancer with small cell carcinoma and mucinous adenocarcinoma of the ovary: a case report and literature review
Source: Front Oncol. 2025 Feb 25;15:1500088. doi: 10.3389/fonc.2025.1500088 (PMC11893415; doi:10.3389/fonc.2025.1500088)
Supplement: Supplementary file 1 [file Table1.docx]

Supplementary Material

# Supplementary Table 1: Clinical features, treatment and prognosis of ovarian small cell carcinoma with mucinous adenocarcinoma

| Case | Age  (years) | | Clinical symptoms | Tumor site | Tumor size  (cm) | Staging | Operation | Chemotherapy | Outcome  (months) |
| --- | --- | --- | --- | --- | --- | --- | --- | --- | --- |
| Jones et al(2) | 65 | Abdominal distention | | LOV | 16.5×13.6×9 | IA | TAH+BSO+OMT+AP | No | Dead (10 months); Liver and peritoneal metastases |
| Collins et al(3) | 34 | Weight loss and abdominal distension | | LOV | 16×11×8 | IC | TAH+BSO+OMT | Yes | Dead (6 months); multiple metastases |
| Khurana et al(4) | 22 | Abdominal pain and pelvic mass | | ROV | NM | IA | RSO+AP | Yes | Dead (3 months); multiple liver metastases |
| Grandjean et al(5) | 32 | Pelvic mass | | LOV | 20×13×11 | IA | BSO+OMT | Yes | Alive |
| Qu et al(6) | 69 | Abdominal protrusion and difficulty urinating | | LOV | 20×13×5 | NM | TAH+BSO | No | Alive |
| Wei et al(7) | 51 | Progressive abdominal distention | | LOV | 13×12×10 | IA | TAH+BSO+OMT | Yes | Dead (8 months); extensive abdominal metastases |
| Mannan et al(8) | 21 | No-symptom | | BOV | 20 | IC | NM | Yes | Dead (10 months) |
| Our case | 24 | Abdominal pain and distension | | LOV | 40×45×30 | IC1 | LSO | No | Dead (11 months) |

TAH: total abdominal hysterectomy; BSO: bilateral salpingo-oophorectomy; RSO: right salpingo-oophorectomy; LSO: left salpingo-oophorectomy; OMT: omentectomy; AP: appendicectomy; LOV: left ovary; ROV: right ovary; BOV: bilateral ovary; NM: Not mentioned.
